# Bacterial biofilm conundrum: insight into the frontiers of antibiotic resistance and state-of-the-art anti-biofilm interventions

**DOI:** 10.3389/fcimb.2026.1589866

**Published:** 2026-02-16

**Authors:** Ayushi Sharma, Poonam Katoch, Rahul Shrivastava

**Affiliations:** 1 Department of Biotechnology and Bioinformatics, Jaypee University of Information Technology, Solan, Himachal Pradesh, India; 2 Department of Biochemistry, Postgraduate Institute of Medical Education and Research, Chandigarh, India; 3 Department of Microbiology, Himachal Pradesh University, Shimla, Himachal Pradesh, India

**Keywords:** AMR, anti-biofilm strategies, antibiotic resistance, biofilms, quorum sensing

## Abstract

Bacterial biofilms are organized multicellular structures enmeshed in a self-secreted extracellular matrix (ECM). The communities present an alarming challenge in the fight against antimicrobial resistance (AMR). They act as a protective niche for microbes, provide chemical and physical protection to the resident cells, allow bacteria to endure host immune responses, and undermine the standard antimicrobial treatments. Despite advancements in microbiological research, biofilms remain an invisible frontier that complicates diagnostics and treatment. This perspective article provides insights into the enigmatic nature of biofilms and examines their role in human infections and diseases. It scrutinizes biofilm AMR mechanisms, including altered metabolic states, ECM-linked decreased antibiotic penetration, and augmented horizontal gene transfer. Further, it delves into the innovative anti-biofilm interventions for mitigating impact of bacterial biofilm on human health. The article also highlights the challenges in engineering ECM for eradicating the recalcitrant biofilms. The article emphasizes critical urgency to integrate biofilm-related research with the comprehensive AMR response, and advocates for interdisciplinary collaborations to transform laboratory discoveries into healthcare advancements. Research uncovering the complexity of biofilms and intriguing therapeutic approaches can address the requirement of revolutionary solutions to combat biofilm-associated infections and ensuing AMR. Overall, this perspective serves as a call to action, underscoring the compelling need to prioritize collective efforts in biofilm research to promote public health.

## 1 Introduction

Biofilms, so called because of their appearance as a slimy layer when visualized through naked eye, were characterized for the first time by Antoni van Leeuwenhoek in 1684 as plaque on his teeth. These were then referred to as ‘animalcules’ (Richards and Ojha, 2014). The term ‘biofilm’ was coined quite later, in 1978, by Costerton and colleagues. These microbial ecosystems have garnered significant scientific attention as more than 99.9% bacteria have been known to efficiently form biofilms on non-living or living surfaces, either on solid platforms, or fluid interfaces (Costerton et al., 1978).

The evidence of bacteria existing as biofilms, which are microbial communities that attach to substrates, is well traced back to fossil records (Decho and Gutierrez, 2017). The extracellular matrix (ECM) rich consortium, comprising extracellular polymeric substances (EPS), not only provides structural integrity but also makes resident cells resistant to environmental stress and antimicrobial agents (Karygianni et al., 2020; Sharma et al., 2023b). They are the outcome of conversion of motile planktonic bacteria to the protective sessile form under extreme environments such as high pressure, variable temperature and pH, oxygen and nutrient limited conditions, and the influence of sub-inhibitory antibiotic concentrations (Yin et al., 2019). The consortium also serve as a protective barrier against desiccation, osmotic shock, the effect of UV light, acids, heavy metals, changing salinity and hydration conditions, altogether boosting the pathogenic and virulence potential of microorganisms (Liu et al., 2023).

Pellicles are another form of biofilms that are observed at the air-liquid interface. This kind is readily observed in bacteria strictly requiring oxygen for their growth. Surface non-requirement for initial bacterial attachment and initiation of colonization makes the pellicles highly advanced in their levels of organization. Moreover, bacterial pellicles have access to nutrients from the liquid medium and oxygen from the air, appearing as a more favorable niche for bacterial survival compared to substrate-attached biofilms (Nait Chabane et al., 2014). Sousa and team demonstrated dense pellicle formation at the air-liquid interface by nontuberculous mycobacteria when compared to biofilms formed by the species at the substrate-liquid interface, highlighting the nature and characteristics of bacterial pellicle biofilms (Sousa et al., 2015).

While the biofilms and pellicles pose challenges in medical and industrial settings, they also hold promise in environmental applications like bioremediation and nutrient cycling (Sharma and Dhiman, 2023). Understanding the environmental, genetic, and molecular factors influencing biofilm formation and behavior is hence pivotal for development of strategies for both mitigating their detrimental impacts and harnessing their beneficial potentials.

## 2 Medical and industrial implications of biofilms

Microbial biofilms are ubiquitous life forms in the environment. They are problematic when linked with pathogenesis and biofouling. Clearance of biofilms from industrial and medical settings is problematic as aversion to the sessile form is the best defense strategy adopted by planktonic bacteria. The US National Institute of Health reports that over 80% nosocomial outbreaks are attributed to bacteria forming biofilm within the human body (Cámara et al., 2022). As per the WHO Global Health Expenditure Database, 2024, global spending on health in 2022 amounted to US$ 9.8 trillion (WHO, 2024), while it was US$ 7.8 trillion concerning healthcare wounds in 2019, of which estimated $281 billion corresponded to biofilm-associated infections (Cámara et al., 2022). Contrarily, addressing the positive impacts, biofilms help in sewage processing (Dhiman and Sharma, 2022), prove beneficial in degrading environmental pollutants, play an important role in sulfur and nitrogen cycles, and carry out fermentation, photosynthesis, and fixation of nitrogen in extreme environments (Cámara et al., 2022).

## 3 Establishment and perpetuation of the bacterial biofilm phenotype

Bacterial biofilm formation is a complex process that progresses through several stages of development, including bacterial adherence to substrates, sessile microcolony formation and biofilm maturation, and biofilm dispersion/detachment which are discussed hereafter. The genetically controlled process helps make the biofilm bacteria phenotypically different from their planktonic counterparts. Biofilm formation requires initial adherence of planktonic bacteria to biotic or abiotic substrates, cell-to-cell communication mediated adhesion between resident bacteria, accumulation of multiple cell layers called ‘microcolony’ in which sessile bacteria produce EPSs (secreted proteins, polysaccharides, extracellular DNA (eDNA), lipids, nucleic acids, and lipoproteins), and the subsequent encapsulation of cell aggregates into an ECM (Muhammad et al., 2020).

### 3.1 Initial surface attachment and irreversible adhesion

Attachment of cells to a solid support can both be reversible or irreversible and involve factors specific to a particular species (Rabin et al., 2015). It initially begins as a chance encounter when planktonic bacteria encounter any surface and cling to it with the assistance of bacterial appendages like pili or flagella, or under the influence of physical forces (Sharma et al., 2023c). Irreversibly adhered bacteria generally exhibit greater tolerance to chemical and physical shearing (Rabin et al., 2015). Hydrophobic interactions, electrostatic forces, and van der Waals interactions are the non-specific physical forces assisting reversible adherence of planktonic cells to substrates, whereas the irreversible adherence is attained through short-range interactions such as ionic, covalent, and hydrogen bonding, and dipole-dipole interactions (Muhammad et al., 2020). The irreversible cell-to-cell adhesion that leads to biofilm formation is additionally mediated by adhesions such as the polysaccharide intercellular adhesion (PIA) which is generated by *Staphylococcus epidermidis* (Sharma et al., 2023c).

### 3.2 Microcolony formation and biofilm maturation

Irreversibly attached and stabilized cells ultimately form sessile microcolonies which are subsequently converted into mature biofilms. Changes in gene expression patterns induce factors necessary for EPS and ECM production and biofilm development (Kostakioti et al., 2013). Chemical signaling (quorum sensing (QS)) allows communication between biofilm-forming cells. Surfactants and phenol-soluble modulins (PSMs) have been shown to play a significant role in the maturation of *Staphylococcal* biofilms through QS-mediated mechanisms (Otto, 2013). Oxygen concentrations, pH, cell density, and nutrient gradients generate heterogeneity in metabolic activities in different locations within mature biofilms. Nutrient concentration usually decreases with increasing depth and distance from the nutrient source, increasing immunity of the resident biofilm-forming cells against conventional antibiotics (Preda and Săndulescu, 2019).

#### 3.2.1 Structural and functional roles of the extracellular matrix

Biofilms are found embedded both in the extracellular polymers secreted by the host including collagen, mucus, and released DNA, and in a self-secreted EPS-rich ECM (Stewart, 2014). The ECM helps maintain rigidity of biofilms, establish the structural integrity of biofilm-forming cells, enables nutrient acquisition, protects the sessile complexes from the effect of antibiotics and disinfectants, and magnifies the virulence of microorganisms. eDNA mediates horizontal gene transfer (HGT) through bacterial conjugation and promotes cell-to-cell association (Karygianni et al., 2020). EPS and ECM production increases under stress conditions. ECM shields bacterial cells from the impact of ultraviolet radiations, changes in pH, provides protection against desiccation and drainage, and help withstand the action of immune effectors (Limoli et al., 2015). ECM imparted high tensile strength allows microbial communities to overcome barriers such as opsonization and phagocytosis (Gunn et al., 2016).

### 3.3 Biofilm dispersal and its implications for infection spread

Certain cells from mature biofilms revert back to the planktonic state with the passage of time. Major factors inducing detachment of cells from bacterial biofilms are QS, declined nutrient levels, shear forces, flow effects, stress induction, oxygen tension, and variable temperature and pH conditions (Muhammad et al., 2020). Detached cells indulge in both the vertical and horizontal transfer of genes and exacerbate the incidences of host infections (Kaplan, 2010). Gene transmission converts non-pathogenic strains into pathogenic strains and enhances bacterial virulence. Since biofilms are notoriously difficult to treat with antibiotics, understanding and disrupting the biofilm dispersal process may reveal novel drug targets for preventing the spread of infections and combating biofilm-associated diseases.

## 4 Determinants of biofilm formation and stability

Substrate adherence relies on a stable availability of oxygen, appropriate temperature, ionic strength, pH, and medium nutrient composition. Substrate hydrophobicity, electrostatic and steric interactions, surface geometry, and the type of biomaterial (smooth, rough, porous, wet, stiff) are other factors responsible for bacterial surface attachment (Zheng et al., 2021). Apart from the substrate properties, the cell surface properties like its charge, cell surface appendages for motility, including glycocalyx, pili, flagella, and fimbriae, the cell surface physiochemical properties like surface hydrophobicity, and the presence of exopolysaccharides and lipopolysaccharides (LPS) are additional crucial factors influencing biofilm formation (Kilic, 2025).

## 5 Cell–cell signaling and regulatory networks in biofilms

Biofilm development is accompanied by the formation of persister cells and QS-mediated gene regulation of processes such as the production of virulent factors, secondary metabolites, and stress adaptation mechanisms (Preda and Săndulescu, 2019).

### 5.1 Quorum sensing–mediated regulation of biofilm development

QS is an intercellular communication mechanism between bacteria that mediates a cell-density dependent synchronized expression of genes. It helps bacteria coordinate metabolism and processes such as biofilm formation, antibiotic recalcitrance, host-microbe symbiosis, HGT, and apoptosis, altogether assisting bacterial adaptation to changing environmental cues. QS is mediated by secretion and accumulation of extracellularly produced inter and/or intracellular signaling molecules named autoinducers, which promote rapid intercellular communication (Juszczuk-Kubiak, 2024). Gram-positive bacteria communicate using the autoinducer peptides, whereas the Gram-negative bacteria predominantly use *N*-acyl homoserine lactone (AHL) molecules (Naga et al., 2023). QS provides an idea of variations in cell density by estimating individual contribution from each biofilm-forming cell, stand responsible for expression of virulent factors, and coordinate host immune responses and biofilm defense mechanisms in environments encountering variable nutrients or toxic substances (Zhang and Powers, 2012). Besides, they regulate biofilm behavior (control adherence of cells to surfaces, matrix synthesis, fluid channel formation, and play a major role in dispersion of sessile cells (Kaplan, 2010).

#### 5.1.1 Two-component system–driven signaling in gram-positive biofilms

Autoinducer peptides are efficiently detected by transmembrane receptors of the two-component system (TCS) on surpassing the threshold concentrations (Paluch et al., 2020). TCS regulates numerous metabolic processes related to biofilm formation such as the cell cycle, intracellular communication, and induction of factors responsible for virulence (Zhang and Powers, 2012). TCS comprises of two proteins, a cytoplasmic response regulator and a membrane-bound sensor (histidine kinase). Donation of phosphate group by ATP to a conserved histidine residue (autophosphorylation) transduces signals which further transfer the phosphate to an aspartate component within the response regulator (differential phosphorylation). The phosphorylated response regulator activates transcription of genes associated with the QS regulon, consequently modulating changes in expression patterns of genes related to biofilm formation (Rutherford and Bassler, 2012).

#### 5.1.2 AHL-dependent signaling in gram-negative biofilms

AHLs are the QS-regulating molecules in Gram-negative bacteria that function with the help of LuxI and LuxR. The autoinducer synthase LuxI biosynthesizes the AHL signal which diffuses out of the bacterial cell, attains a critical threshold (with increasing cell density), binds to the DNA-binding transcription activator (LuxR receptor), and activates the expression of biofilm-associated genes (Zhou et al., 2020).

### 5.2 Cyclic dimeric guanosine monophosphate signaling and phenotypic switching

The second messenger cyclic dimeric guanosine monophosphate (c-di-GMP) also contributes towards formation and stabilization of biofilms by promoting producing of adhesion proteins, modulating bacterial motility rates, and enhancing virulence (Liu et al., 2022). Its functional mechanism involves binding with several receptors like riboswitches, adaptor proteins, transcription factors, and enzymes (Roy et al., 2018). Elevated c-di-GMP levels boosts synthesis of extracellular appendages and exopolymers (Palmer and Stoodley, 2007). It even plays an important role in QS phenomenon by facilitating phenotypic transitions between planktonic and sessile forms, interceding cellular differentiation, and mediating bacterial persistence and pathogenicity (Sharma et al., 2014).

## 6 Antimicrobial resistance in bacterial biofilms

Antibiotics used for growth promotion in livestock, domesticated animals, and agriculture screen back bacteria alarmingly resistant to antibiotics (Kumar et al., 2022; Zhang et al., 2024). The increased AMR called recalcitrance causes accumulation of biofilms on medical devices such as prosthetic heart valves, contact lenses, orthopedic devices, urethral and intravascular catheters; increasing the load of medical infections (Mishra et al., 2024). Biofilm formation has been clinically linked to numerous bacterial species, with the most significant human pathogens responsible for biofilm-associated infections summarized in **Table 1**.

**Table 1:** Biofilm-associated bacterial pathogens: clinical manifestations and mechanisms of resistance to antibiotics and host defenses.

Biofilm-forming bacterial species	Associated infections	Mechanisms of antibiotic and immune evasion	Reference
*Staphylococcus aureus*	Endocarditis, bacteremia, prosthetic joint infections, skin infections	Biofilm formation, expression of efflux pumps, alteration of penicillin-binding proteins, formation of small colony variants (SMVs)	(Zhou et al., 2022; Pai et al., 2023)
*Mycobacterium tuberculosis*	Tuberculosis	Biofilm and pellicle formation, efflux pumps, cell wall impermeability, cell wall structure alteration, acquisition of gene mutations conferring drug resistance, dormancy	(Assefa and Girmay, 2024)
*Staphylococcus epidermidis*	Catheter- and indwelling medical device-related infections	Biofilm formation, presence of ECM called glycocalyx or slime, production of PIA	(Chen et al., 2019)
*Klebsiella pneumoniae*	Pneumonia, urinary tract infections (UTIs), bloodstream infections	Biofilm formation, capsule and LPS production, expression of fimbriae for adhesion, carbapenemase production, efflux pumps	(Pai et al., 2023)
*Pseudomonas aeruginosa*	Cystic fibrosis lung infections, ventilator-associated pneumonia, systemic infections	Biofilm formation, production of efflux pumps (e.g., MexAB-OprM and MexXY-OprM), β-lactamase production, QS-mediated resistance	(Haidar et al., 2024)
*Acinetobacter baumannii*	Ventilator-associated pneumonia, catheter-related infections, UTIs, bloodstream and wound infections	Biofilm formation, efflux pumps, modifications of target sites, production of β-lactamases, desiccation resistance	(Gedefie et al., 2021)
*Escherichia coli*	Urinary tract infections, catheter-associated infections, dysbiosis	Biofilm formation, expression of fimbriae for adhesion, production of extended-spectrum β-lactamases (ESBLs), upregulation of efflux pumps	(Pai et al., 2023)
*Staphylococcus haemolyticus*	Bloodstream infections, chronic prostatitis, ocular infections, wound infections, UTIs	Biofilm formation, QS, production of antibiotic-degrading enzymes	(Eltwisy et al., 2022)
*Enterococcus faecalis*	Root canal infections, UTIs, endocarditis	Biofilm formation, intrinsic resistance to many antibiotics, ability to survive harsh conditions, and production of cytolysin, gelatinase, hyaluronidase, and serine proteases	(Fiore et al., 2019)
*Listeria monocytogenes*	Listeriosis, meningitis, bacteremia, encephalitis, sepsis	Biofilm formation, formation of persister cells, presence of mobile genetic elements (plasmids and transposons), intracellular survival, resistance to environmental stresses	(Matereke and Okoh, 2020)
*Stenotrophomonas maltophilia*	Respiratory tract infections, bloodstream infections	Biofilm formation, use of motility, iron acquisition mechanisms, production of metallo-β-lactamases, efflux pumps, outer membrane components, protein secretion systems	(Brooke, 2021)
*Enterococcus faecium*	UTIs, bloodstream infections, endocarditis	Biofilm formation, vancomycin resistance, efflux pumps	(Fiore et al., 2019)
*Streptococcus pneumoniae*	Otitis media infections, sinusitis, pneumonia, meningitis, bacteremia	Biofilm formation, alteration of penicillin-binding proteins, production of capsule, efflux pumps	(Brooks and Mias, 2018)
*Burkholderia cenocepacia*	Lung infections in cystic fibrosis patients, bloodstream infections	Biofilm formation, efflux pumps, resistance to disinfectants, production of exopolysaccharides and QS-controlled virulence factors	(Ganesh et al., 2020)
*Streptococcus pyogenes*	Pharyngitis, cellulitis, impetigo, necrotizing fasciitis, puerperal sepsis, myositis, toxic shock syndrome	Biofilm formation, production of streptolysins, presence of a hyaluronic acid capsule, increasing macrolide resistance	(Young et al., 2022)
*Proteus mirabilis*	Catheter-associated UTIs, bacteremia, urosepsis, formation of urinary stones (urolithiasis)	Biofilm formation, urease production leading to stone formation, swarming motility, fimbriae and other adhesins, iron and zinc acquisition, proteases and toxins	(Schaffer and Pearson, 2017)
*Staphylococcus pseudintermedius*	Skin and surgical site infections, catheter-related infections, rhinosinusitis	Biofilm formation, methicillin resistance, zoonotic transmission potential	(Moses et al., 2023)
*Helicobacter pylori*	Gastric ulcers, gastritis, duodenal ulcer, gastric cancer	Biofilm formation, efflux pumps, urease and ferritin production, involvement of outer membrane vesicles (OMVs) in mutation and HGT	(Fauzia et al., 2024)
*Salmonella* species (*S. enteric* and *S. bongori*)	Gastroenteritis, typhoid fever, bacteremia	Biofilm formation, efflux pumps, resistance to bile salts, survival in macrophages, resistance to antimicrobial peptides	(Pradhan and Negi, 2019)
*Enterobacter cloacae*	UTIs, respiratory tract infections, skin and soft-tissue infections, bacteremia, post-neurosurgical meningitis	Biofilm formation, production of ESBLs	(Salimiyan Rizi et al., 2020)

**Figure 1:**
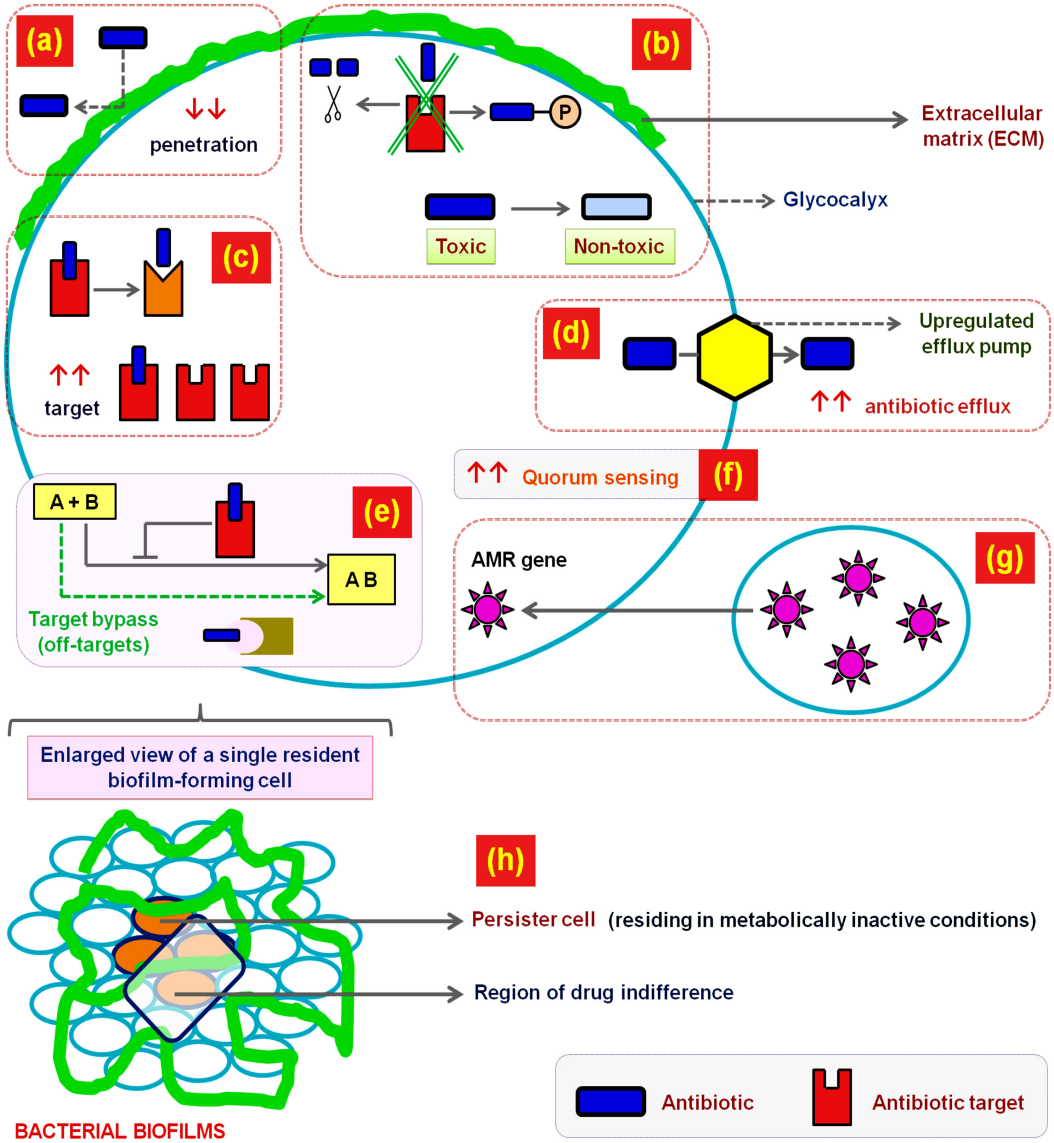
Mechanisms of antibiotic resistance in bacterial biofilms. The figure includes an enlarged view of a single resident biofilm-forming cell within a biofilm, emphasizing the structural complexity and spatial heterogeneity that support biofilm resilience and survival in hostile environments, such as antibiotic exposure. It illustrates the multifaceted mechanisms employed by bacterial biofilms to resist antibiotic action: **(a)** Impaired antibiotic penetration: The ECM or glycocalyx surrounding biofilm-forming cells acts as a physical and chemical barrier to antibiotics. The matrix may prevent antibiotics from reaching their target by trapping them through charge-based interactions or steric hindrance, reducing drug penetration (indicated by downward red arrows). **(b)** Enzymatic inactivation of antibiotics: Biofilm bacteria can produce enzymes that may either destroy or modify antibiotics. This includes enzymatic cleavage, inactivation/modification via phosphorylation, and conversion of toxic antibiotics to non-toxic forms, neutralizing their effect before reaching bacterial targets. **(c)** Target modification: Bacteria evade antibiotic action by modifying the drug’s target site or increasing target abundance (upregulated target production). **(d)** Efflux mechanisms: Upregulated efflux pumps actively expel antibiotics from the bacterial cell, decreasing intracellular drug concentration. **(e)** Metabolic pathway modulation and target bypass: Some biofilm bacteria alter their metabolic pathways to bypass the effects of certain inhibitors, utilize alternative substrates, and even turn to “off-target” metabolic pathways for survival, ensuring uninterrupted metabolic flow despite the presence of antibiotics. **(f)** Quorum sensing and cell communication: The bacteria resident within biofilms engage in quorum sensing (QS)-a density-dependent signaling system which coordinates expression of genes responsible for antibiotic resistance, efflux pumps, and metabolic modulation. **(g)** Horizontal gene transfer (HGT): The close spatial proximity of cells in biofilms promotes the exchange of mobile genetic elements, including antibiotic resistance genes, through HGT. This sharing contributes to the emergence and clonal expansion of drug-resistant populations within the biofilm. **(h)** Metabolic dormancy and persister cells: Within biofilms, nutrient and oxygen gradients lead to zones of slow-growing or metabolically inactive cells. Persister cells reside in regions of drug indifference and are tolerant to antibiotics targeting active growth processes. These persisters contribute significantly to treatment failure and biofilm recalcitrance.

Possible explanations to the ever increasing AMR have been summed up under the following: upregulation of efflux pumps that consequently decrease antibiotic concentration within bacteria (Barnabas et al., 2022; Katoch et al., 2024), presence of glycocalyx, degradation or inactivation of antibiotics by enzymes conferring neutralization potentials like hydrolysis or modification, dense ECM acting as an antibiotic penetration barrier either by reacting chemically with the drug molecules or by limiting their diffusion, transport, or target recognition potential, changes in the mechanisms of QS, and the presence of dormant antibiotic protected non-dividing bacterial population within sessile conjugates called persister cells (Lebeaux et al., 2014; Belay et al., 2024) (**Figure 1**).

HGT is a major contributor to global AMR crisis as cells within a biofilm are immobilized and tightly packed, promoting intense cell-to-cell interactions that make biofilms highly conducive to HGT. AMR genes in such conditions spread through several pathways, including conjugation mediated through conjugative plasmids, as well as integrative and conjugative elements, and transduction via bacteriophages, that help biofilms establish as a reservoir of resistance genes (Liu et al., 2024). Recent studies have, however, even proposed alternate mechanisms of HGT, including lateral transduction (LT) and OMV-mediated transfer. LT, the process in which large sections of the bacterial genome are mobilized by temperate bacteriophages has been reported to facilitate transfer of *S. aureus* pathogenicity islands (SaPIs), large mobile gene clusters that code for various accessory proteins, and virulence factors, to new bacterial cells (Chee et al., 2023). Additionally, OMVs obtained from *P. aeruginosa* biofilms were shown to horizontally transfer the plasmid DNA more efficiently compared to those obtained from planktonic populations, eventually leading to dissemination of AMR genes within and between bacterial species (Johnston et al., 2023).

In addition, several other factors also contribute to the AMR offered by biofilms. These include bacterial adaptation to sub-lethal drug or disinfectant concentrations, metabolic activities undergoing within cell aggregates and the phenotypic differences between them, indifference in drug permeability regions, expression of genetic mechanisms specific to biofilms, reduction in antibiotic metabolism, modulation of metabolic processes and stress responses by resident biofilm bacteria, and antibiotic target modifying events such as mutations or the existence of an alternate gene (Lebeaux et al., 2014; Otter et al., 2015).

## 7 Persistence and phenotypic heterogeneity in bacterial biofilms

Persisters are a drug-susceptible quiescent form of bacteria that have the potential to survive under stress conditions, such as reactive oxygen species, acid pH, starvation, and antibiotics (Niu et al., 2024). Bacterial persisters were identified around mid-1900s by Joseph Bigger within a population of planktonic *S. aureus* (Bigger, 1944). They were, however, reported for the first time by Spoering and Lewis. The bacterial population existing as persisters is typically found within completely protective states and displays increased resistance towards drugs (Spoering and Lewis, 2001). Switching of phenotypes from planktonic to sessile forms slows down bacterial growth and either decreases or completely inhibits the activity of antibiotics. Lack of nutrients also causes the metabolically inefficient slow or non-growing resident biofilm bacteria attribute to persistence (Kostakioti et al., 2013). Moreover, biofilm heterogeneity favors the formation of persister cells. Although genetically similar to their planktonic counterparts, cells residing within biofilms help the persister cells tolerate even the bactericidal drug concentrations (Liu et al., 2024).

Accumulating evidence indicates that, biofilm-associated persistence is strongly influenced by spatial and physiological heterogeneity arising from steep gradients in nutrients, oxygen, pH, and signaling molecules across the biofilm architecture. Cells located in deeper biofilm layers experience nutrient deprivation and hypoxic or anoxic conditions, leading to reduced metabolic activity and diminished antibiotic susceptibility, particularly to agents targeting actively dividing cells. Additionally, QS signals and stress-response pathways are unevenly distributed within biofilms, further reinforcing localized phenotypic diversification and promoting the formation of persister subpopulations. Such microenvironment-driven heterogeneity enables biofilms to function as structured communities rather than uniform cell populations, thereby contributing to treatment recalcitrance and infection chronicity (Flemming et al., 2016; Sauer et al., 2022).

Phenotypic heterogeneity, which includes variation in persistence levels, metabolic diversity, and differences in size of bacterial colonies which is reflected in SMVs, increasingly makes the persisters distinct from drug-tolerant and drug-resistant bacteria. However, the persisters and drug-resistant bacteria are not completely unrelated as they may interconvert and overlap under specific conditions. In the present scenario, tolerance of persisters to various drugs remains underexplored, although the mechanism has been investigated to be different from that of conventional bacterial resistance, while sharing certain features like enhanced efflux and decreased antibiotic penetration (Niu et al., 2024).

An additional focus in persister research is the toxin-antitoxin (TA) system and its role in mediating phenotypic switching within biofilms. TA systems, the genetic elements present in bacterial cells that encode for a toxin and its corresponding antitoxin, which were initially linked to maintenance of plasmids during cell division, are being linked with bacterial drug resistance and the formation of persisters (Kunnath et al., 2024). On antibiotic exposure, bacteria with certain TAs have the ability to enter a dormant or persister state which allows their survival until the exposure prolongs (Harms et al., 2016). TAs have also been shown to induce persister formation by escalating a state of reversible growth arrest in bacterial cells, allowing cells to conserve energy and withstand environmental stresses, including antibiotic treatment, eventually augmenting the disseminating and maintenance of antimicrobial resistance (Yang and Walsh, 2017).

## 8 Biofilm-associated human infections and clinical burden

Bacterial biofilms contribute to about 65% and 80% microbial and chronic infections, respectively, owing to microbial ability to accumulate on wounds, endoscope tubes, and medical devices (Jamal et al., 2018; Sharma et al., 2021; Perry and Tan, 2023). The first report linking biofilm and infections associated with implanted devices was published in the year 1982. The study was concerned with electron microscopy of a pacemaker lead in a patient suffering from recurring *S. aureus* bloodstream infection (Marrie et al., 1982; Lebeaux et al., 2014).

Medical devices contaminated with microbial biofilms usually cause infections in humans. Medical implants include urinary catheters, prosthetic joints, contact lenses, mechanical heart valves, central venous catheters, voice prostheses, pacemakers, peritoneal dialysis catheters, ventricular shunts, and mechanical heart valves (Jamal et al., 2018). Biofilms also play a vital role in chronic infections like cystic fibrosis pneumonia, periodontitis, bacterial prostatitis, endocarditis, chronic otitis media, infections from burn wound, osteomyelitis, and rhinosinusitis (Perry and Tan, 2023).

Mechanisms initiating host-biofilm infections include endotoxin production, detachment of aggregates from medical implants (triggers urinary tract or bloodstream infections), inherent resistance to the host immune responses, and exchange of AMR plasmids (Donlan and Costerton, 2002). Additionally, the matrix encompassing tenacious biofilms decreases host immunity by acting as a filter that entraps minerals and host serum components (platelets, RBCs, and fibrin) (Donlan, 2001). Worsening the scenario, biofilm antigens continuously generate antibodies which damage the surrounding host tissues, apart from the damage caused by the existent cell aggregates (Chadha, 2014).

## 9 Therapeutic strategies targeting biofilms: limitations and opportunities

**Figure 2:**
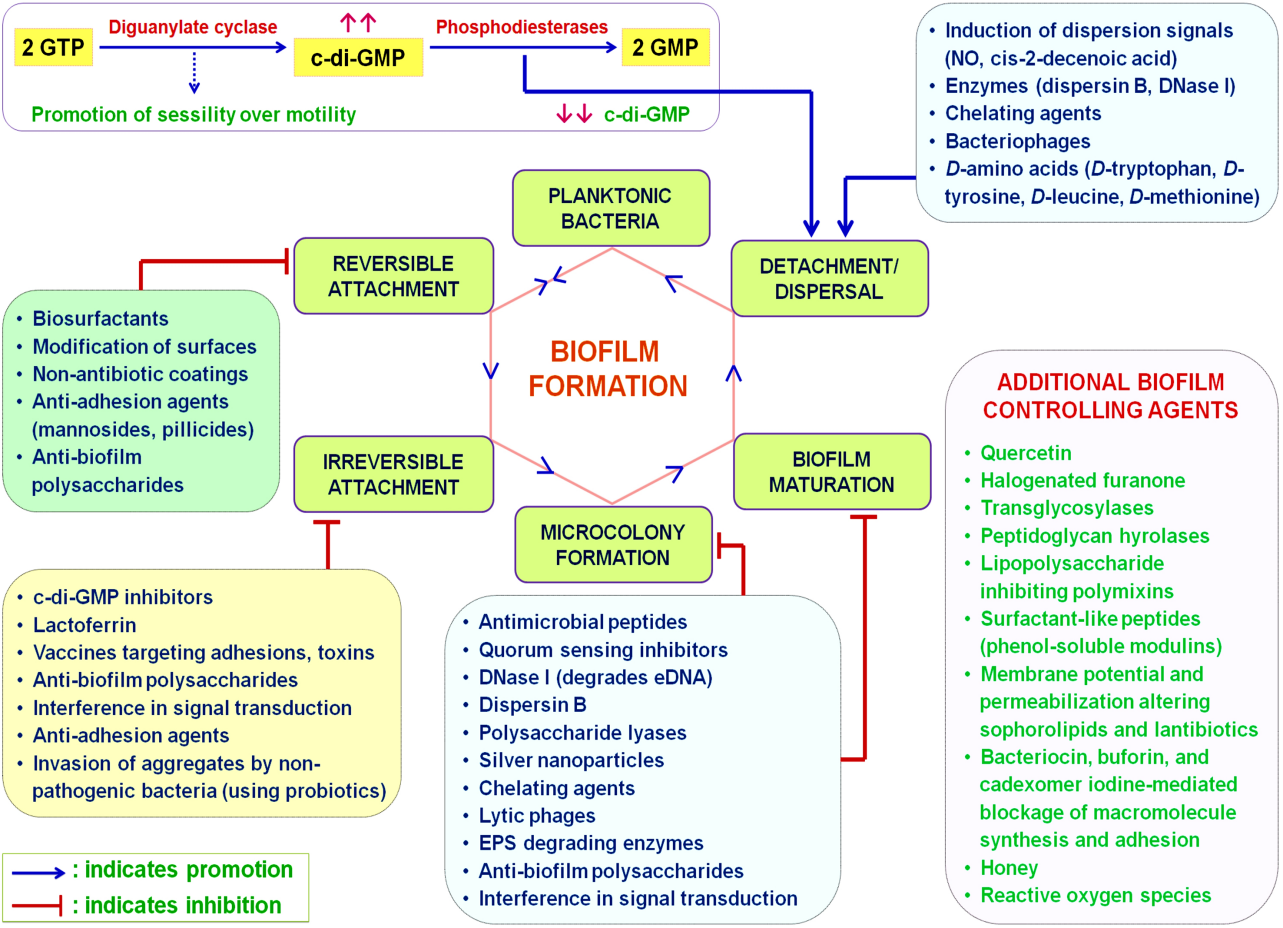
Bacterial biofilm inhibition strategies. Initial phases of biofilm development, including reversible and irreversible attachment, can be inhibited by preventing the attachment of microorganisms, whereas later steps, such as microcolony formation, biofilm maturation, and dispersal, can be controlled by manipulating the production of EPS and signaling molecules.

Increased prevalence of opportunistic pathogens exhibiting a biofilm-forming tendency infects a wide range of hosts, leads to AMR, prolonged persistence, and robustness towards eradication (Sharma et al., 2022a; Sharma et al., 2022b; Sharma et al., 2023a). Rapid-action treatments facilitating clearance of biofilms from both the environment and infected hosts are therefore required (Kostakioti et al., 2013). Increasing the concentration of antibiotics and disinfectants causes environmental damage, leads to emergence of multi-drug resistance instead of sharing a helping hand, and boosts hospital-related infection rates. Magnifying health concerns demand the development of novel biofilm-inhibition strategies (Lebeaux et al., 2014). **Figure 2** outlines compounds with potential sessile bacterial complex eradication ability. Despite their general resistance to disinfection, biofilms can be inhibited by various disinfectants, including hydrogen peroxide, benzalkonium chloride, and chlorhexidine (Otter et al., 2015). Contradictorily, antiseptics and biocides chlorhexidine and glutaraldehyde cross-link with biofilms, subsequently aiding bacterial aggregation (Stewart, 2014). However, modifications in c-di-GMP pathway and inhibition of bacterial QS have shown promising results in reducing human infections related to biofilms (Roy et al., 2018).

Of particular note, advances in synthetic biology have enabled the development of engineered bacteriophages that express biofilm-degrading enzymes during infection, thereby simultaneously targeting bacterial cells and the ECM, resulting in markedly enhanced biofilm disruption compared with non-enzymatic phage treatments (Lu and Collins, 2007). More recently, nanotechnology- and CRISPR-based approaches have emerged as promising additions to the anti-biofilm arsenal. Nanoparticle-based systems, including metal nanoparticles, polymeric nanoparticles, and lipid-based nanocarriers, have demonstrated enhanced penetration into biofilms, improved local drug retention, and the ability to co-deliver antimicrobials with matrix-disrupting or QS-inhibitory agents (Huh and Kwon, 2011; Sedighi et al., 2024). These platforms may be engineered to respond to biofilm-specific cues such as pH, enzymatic activity, or redox conditions, which may improve targeting efficiency while potentially reducing systemic toxicity. In parallel, CRISPR-Cas-based antimicrobials also offer a highly specific strategy to selectively eliminate biofilm-associated pathogens or resistance determinants by sequence-directed genome targeting. While proof-of-concept studies demonstrate effective disruption of biofilms and removal of resistance genes *in vitro* and in animal models, significant challenges remain with the used of CRISPR antimicrobials, particularly related to delivery efficiency, off-target effects, microbial escape, and regulatory feasibility (Gager and Flores-Mireles, 2024; Rahman et al., 2025; Saffari Natanzi et al., 2025). Taken together, bacteriophage therapies, nanotechnology-enabled platforms, and CRISPR-based antimicrobials remain largely at a preclinical stage, highlighting the need for continued optimization and rigorous translational validation prior to clinical implementation.

### 9.1 Challenges in engineering the biofilm ECM for effective control

Engineering the biofilm ECM as a therapeutic target remains challenging due to its dynamic, heterogeneous, and species-specific composition, which limits the universal applicability of ECM-disrupting strategies. The ECM is a highly heterogeneous and adaptable structure, the composition and abundance of whose components, vary not only between species but also across environmental conditions and infection sites, limiting the utility of single-target interventions (Flemming et al., 2016; Karygianni et al., 2020). Enzymatic degradation strategies, such as DNases or polysaccharide hydrolases, demonstrate promising biofilm disruption *in vitro*, however, their clinical translation is constrained by poor stability *in vivo*, including susceptibility to denaturation, proteolytic degradation, and rapid systemic clearance (Thorn et al., 2021). Moreover, biofilm dispersal, while central to bacterial transmission between environments and hosts, may be unintentionally promoted by partial ECM disruption, as it leads to the release of highly tolerant bacterial cells capable of reseeding infection and exacerbating disease spread (Kaplan, 2010). The adaptive capacity of biofilms further complicates ECM engineering, as bacteria can remodel matrix composition in response to selective pressure and environmental cues, thereby diminishing long-term efficacy of single-target interventions (Flemming and Wingender, 2010). These challenges underscore the need for context-specific and multifunctional ECM-targeting strategies, ideally integrated with antimicrobials or immune-modulatory approaches, rather than reliance on matrix degradation alone.

### 9.2 Context-specific and integrated ECM-targeting strategies

Constraints and limitations associated with enzymatic degradation of biofilm ECM components underscores the need for integrated and context-specific ECM-targeting strategies that go beyond enzyme selection alone and instead focus on optimizing delivery, stability, and site-specific activity. In this regard, advanced drug delivery platforms have emerged as emerging technologies, offering encapsulation and stabilization of biofilm-dispersing enzymes, protection them from hostile physiological environments, and preservation of their enzymatic bioactivity. In particular, smart and bio-responsive delivery systems enable targeted, site-specific release of enzymes at infected niches, improving therapeutic efficacy while minimizing off-target effects and systemic exposure (Thorn et al., 2021).

Combinatorial strategies that couple ECM-disrupting agents with conventional antibiotics have demonstrated enhanced antimicrobial penetration and improved killing of biofilm-embedded bacteria, particularly when enzymatic degradation of eDNA or polysaccharides is synchronized with antibiotic exposure (Aung et al., 2016). Beyond antibiotics, integration of ECM-targeting approaches with immune-modulatory strategies may offer additional promise, as biofilm matrices actively shield bacteria from phagocytosis and dampen host immune signaling (Gunn et al., 2016). However, the efficacy of such integrated strategies is highly context dependent, varying with infection site, host immune status, and biofilm composition (Van Roy and Kielian, 2025), underscoring the need for tailored therapeutic designs rather than universal anti-biofilm solutions. The insights hence support a shift toward synergistic, systems-level anti-biofilm therapies that combine ECM disruption, antimicrobial action, and host immune engagement to overcome the resilience of chronic biofilm-associated infections.

## 10 Critical appraisal of biofilm resistance mechanisms and translational readiness of anti-biofilm strategies

Although multiple mechanisms have been proposed to explain biofilm-associated antimicrobial resistance, they are not equally supported by current experimental and clinical evidence. Robust data consistently support the roles of physiological heterogeneity, metabolic dormancy, persister cell formation, and restricted antimicrobial penetration within the ECM as dominant contributors to biofilm tolerance across diverse bacterial species and infection models (Flemming et al., 2016; Liu et al., 2024). In contrast, other mechanisms, particularly HGT within biofilms, appear to be highly context dependent, with their contribution varying according to species, biofilm architecture, and environmental conditions (Michaelis and Grohmann, 2023). While OMVs have been shown to transport DNA, resistance determinants, and signaling molecules *in vitro* (Johnston et al., 2023; Fauzia et al., 2024), direct evidence that OMV-mediated gene transfer constitutes a major driver of antimicrobial resistance under natural or clinical infection conditions remains limited. Similarly, anti-biofilm interventions differ markedly in their translational maturity.

Strategies such as QS inhibition, enzyme-assisted ECM disruption, and select nanotechnology-enabled delivery systems demonstrate increasing preclinical and early translational promise, supported by growing *in vivo* validation and improved understanding of biofilm-specific vulnerabilities. These approaches aim to weaken biofilm structural integrity or interfere with coordinated bacterial behavior, thereby enhancing susceptibility to antimicrobial agents and host immune defenses. In contrast, emerging modalities including CRISPR-based antimicrobials and engineered bacteriophage therapies, while conceptually transformative, largely remain at an experimental stage. Their clinical translation is constrained by unresolved challenges related to efficient and targeted delivery within complex biofilm environments, narrow host specificity, potential off-target effects, and regulatory considerations associated with genetically programmable or replicating biological agents. In light of these disparities, a critical appraisal of evidence strength and translational readiness is essential to prioritize biologically relevant mechanisms and therapeutically actionable strategies, thereby guiding future investment and research efforts in anti-biofilm therapeutics.

## 11 Concluding remarks and future directions

Biofilms are 3D structures that act as microbial battlefront under extreme environmental conditions, facilitated by the production of ECM and QS-mediated cell signaling. Bacteria biofilms have varied implications in medical, industrial, and environmental sectors. While biofilms cause biofouling, AMR, pathogenesis, and virulence, they also hold potential for degradation of organic pollutants, owing to the provision of a low-budget, eco-friendly, and green technology (Ali et al., 2023; Lago et al., 2024).

Biofilms formed on indwelling medical implants contribute towards persistent and recurrent infections, aggravating the global AMR crisis. The standard antibiotics are predominantly developed for planktonic bacteria and often appear inefficient against biofilms. This necessitates the development of novel anti-biofilm strategies. However, despite decades of ongoing research, the complexities of biofilm formation, maintenance, and dispersion have still not been completely explored. Future research should, therefore, focus on elucidation of intricate molecular mechanisms behind biofilm formation and resistance, emphasizing the development of targeted anti-biofilm strategies.

Some promising biofilm combating approaches encompass the utility of QS inhibitors, biofilm-disrupting agents, phage therapy, ECM degrading enzymes, and gene editing tools concerning CRISPR-Cas systems. In addition, cutting-edge drug delivery systems, such as nanoparticles, may serve as conveyers of agents targeting biofilm EPS owing to their large surface area-to-volume ratio, small size, and high sensitivity, making them exceptionally suitable for penetrating and destroying biofilms (Eghbalpoor et al., 2024). Research delving into collective genomics, transcriptomics, and proteomics approaches could also provide a thorough understanding of the regulatory and signaling pathways underlying biofilm formation. Apart from this, the emerging novel therapeutic interventions still require further exploration for effective confrontation of biofilm-associated infections.

From an industrial perspective, the use of beneficial bacterial biofilms to prevent corrosion, development of non-fouling materials, and modification of surfaces that inhibit the formation of biofilms could cut-down operational challenges and maintenance costs. In environmental science, leveraging unreported biofilm-forming bacteria for applications like wastewater treatment, degradation of pollutants, and inhibition of corrosion may continue to provide substantial benefits to the living population (Muhammad et al., 2020).

Furthermore, the discovery of biofilm-targeting drugs and bioengineering solutions may be accelerated by integrating experimental studies with bioinformatics and artificial intelligence. Interdisciplinary collaborations across academia, microbiology, computational biology, materials science, industry, and healthcare may also address the dual challenges of mitigating the adverse impacts of biofilms while harnessing their beneficial abilities.

Overall, a comprehensive understanding of biofilm biology, combined with innovative technologies, will pave the way for novel strategies to confront biofilm-related challenges and unlock their potential in medicine, industry, and environmental applications.

## Data Availability

The datasets presented in this article are not readily available because No dataset has been used. Requests to access the datasets should be directed to juit.rahul@gmail.com.
